# Glowing Sucker Octopus (*Stauroteuthis syrtensis*)‐Inspired Soft Robotic Gripper for Underwater Self‐Adaptive Grasping and Sensing

**DOI:** 10.1002/advs.202104382

**Published:** 2022-04-07

**Authors:** Mingxin Wu, Xingwen Zheng, Ruosi Liu, Ningzhe Hou, Waqar Hussain Afridi, Rahdar Hussain Afridi, Xin Guo, Jianing Wu, Chen Wang, Guangming Xie

**Affiliations:** ^1^ State Key Laboratory for Turbulence and Complex Systems College of Engineering Intelligent Biomimetic Design Lab Peking University Beijing 100871 P. R. China; ^2^ Advanced Production Engineering, Engineering and Technology Institute Groningen Faculty of Science and Engineering University of Groningen Groningen 9747AG The Netherlands; ^3^ Department of Bioengineering Imperial College London South Kensington London SW7 2AZ UK; ^4^ School of Aeronautics and Astronautics Sun Yat‐Sen University Guangzhou 510006 P. R. China; ^5^ Peng Cheng Laboratory Shenzhen 518055 China; ^6^ Institute of Ocean Research Peking University Beijing 100871 China; ^7^ Southern Marine Science and Engineering Guangdong Laboratory (Guangzhou) Guangzhou 511458 P. R. China

**Keywords:** bioinspiration, glowing sucker octopus, self‐adaptive grasping, soft gripper

## Abstract

A soft gripper inspired by the glowing sucker octopus (*Stauroteuthis syrtensis*)’ highly evolved grasping capability enabled by the umbrella‐shaped dorsal and ventral membrane between each arm is presented here, comprising of a 3D‐printed linkage mechanism used to actuate a modular mold silicone‐casting soft suction disc to deform. The soft gripper grasp can lift objects using the suction generated by the pump in the soft disc. Moreover, the protruded funnel‐shaped end of the deformed suctorial mouth can adapt to smooth and rough surfaces. Furthermore, when the gripper contacts the submerged target objects in a turbid environment, local suctorial mouth arrays on the suction disc are locked, causing the variable flow inside them, which can be detected as a tactile perception signal to the target objects instead of visual perception. Aided by the 3D‐printed linkage mechanism, the soft gripper can grasp objects of different shapes and dimensions, including flat objects, objects beyond the grasping range, irregular objects, scattered objects, and a moving turtle. The results report the soft gripper's versatility and demonstrate the vast application potentials of self‐adaptive grasping and sensing in various environments, including but are not limited to underwater, which is always a key challenge of grasping technology.

## Graphical abstract

A soft gripper inspired by the glowing sucker octopus’ enhanced grasping capability enabled by the umbrella‐shaped dorsal and ventral membrane between each arm is elaborated here. The gripper features excellent abilities to suck and grasp objects with any shapes (planar and nonplanar) and dimensions. Furthermore, the gripper could generate tactile sensation for turbid water environment exploration.

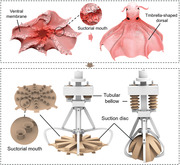

## 1 Introduction

The 2011 U.S. Bureau of Labor Statistics recorded that divers were subject to 38 times the average national occupational death rate (about 3.5 in 100 000)^[^ ^1^ ^]^ because the long‐period underwater tasks would cause severe decompression illness.^[^ ^2^ ^]^ Underwater fishing is one of the typical tasks for divers. Using a robotic gripper to replace divers can reduce the health harm to divers and improve the underwater operation's efficiency.^[^ ^3^ ^]^ However, the robotic gripper with self‐adaptive sensing and grasping ability to target objects with various dimensions and shapes is always challenging. Traditional mechanical grippers usually use rigid contact to grasp the target object to complete a single task,^[^ ^4^ ^]^ causing difficulty grasping irregular objects.^[^ ^5^ ^]^ In recent years, more attention has been paid to studying robotic grippers with a self‐adaptive grasping ability.^[^ ^6^ ^]^ Considering a fingers‐like robotic gripper employing soft fingers actuated by the air from multiple separated air chambers to form the fingers arrays of a robotic gripper, thus increasing the dexterity (degree of freedom) of fingers‐like gripper relative to utilizing rigid joint structures,^[^ ^7^ ^]^ thereby improving the gripper's adaptability to objects with various dimensions and shapes.^[^ ^8^ ^]^ Furthermore, materials with adjustable stiffness were validated to improve the robotic gripper's load capacity significantly.^[^ ^9^, ^10^ ^]^ In addition, various actuation methods, including pump actuation, chemical stimulation,^[^ ^11^ ^]^ electromagnetic,^[^ ^12^, ^13^ ^]^ electroactive polymers,^[^ ^14^, ^15^ ^]^ and thermal or light reactions, enabled the robotic gripper's vast applications in various environments.^[^ ^16^, ^17^ ^]^

However, due to the fingers’ fixed bending mode and the discrete distribution, typically, there was a gap between the target object and the object during contact, which may affect grasping stability. A gripper, which imitates the movement of the sea anemone's outer skin rolling inward to squeeze and wrap the target, can adaptively wrap objects with various shapes and dimensions, thus improving grasping stability.^[^ ^18^ ^]^ However, such a gripper has the limitation of grasping scattered objects, flat objects, and objects beyond the grasping range. In contrast, a fluid‐driven fingerless compliant grippe can generate various grasping modes to adapt to the object being grasped.^[^ ^19^ ^]^ However, its suction mode is limited to smooth surfaces. It is especially worth mentioning that the suction mechanism is a common adhesion system and is widely used in robots. Various attempts have been made to achieve strong suction on rough surfaces and irregular 3D geometries. Tramacere et al. proposed the first artificial sucker inspired by the octopus's sucker.^[^ ^20^ ^]^ Baik et al. achieved wet‐tolerant adhesive by microfabricating the surface of artificial octopus suction cups.^[^ ^21^ ^]^ Inspired by the northern clingfish (Gobiesox maeandricus), Sandoval et al. designed an artificial suction cup that exhibited adhesion on rough surfaces and nonplanar geometries.^[^ ^22^ ^]^ The suction cup developed by Song et al. has an adaptive self‐sealing capability while achieving superior 3D geometrical adaptability and high suction power.^[^ ^23^ ^]^ However, only the suction mode will affect the versatility of the grasping hand.

On the other hand, to make the gripper more controllable, safer, and self‐adaptive to various target objects (different dimensions and shapes) while interacting with the environment or humans, it is worth studying the perception ability of the robotic gripper to the target object,^[^ ^24^ ^]^ especially in the absence of visual perception in a turbid underwater environment. Sensors, including resistance sensors,^[^ ^25^ ^]^ capacitive sensors,^[^ ^26^ ^]^ piezoelectric sensors,^[^ ^27^ ^]^ and optical sensors,^[^ ^28^ ^]^ were typically used in robotic grippers to sense target objects.^[^ ^6^ ^]^ For example, the self‐powered triboelectric nanogenerator^[^ ^29^ ^]^ was combined with a fingered gripper to determine the bending position^[^ ^30^ ^]^ and bending angle^[^ ^31^ ^]^ based on the voltage variation in the circuits, thus inferring the object's dimension.

Many animals can grasp target objects by using their soft tissues,^[^ ^32^ ^]^ which inspires the development of robotic grippers. The dorsal and ventral membranes cover most of the arm's length of the glowing sucker octopus (*Stauroteuthis syrtensis*) and Vampire squid, forming a unique umbrella shape,^[^ ^33^ ^]^ unlike most octopuses with only flexible arms with adhesive suckers distributed on the surface. Such a feature can effectively increase the grasping reliability when gasping large object and living animal by preventing the animal from escaping from the gap between the arms and increasing the grasping friction force.^[^ ^34^, ^35^ ^]^ In this study, inspired by the predation behavior of the glowing sucker octopus (*Stauroteuthis syrtensis*), and benefited from 3D printing and casting technology, we developed a novel soft gripper with a similar geometric structure and grasping ability to the glowing sucker octopus (*Stauroteuthis syrtensis*). The gripper features excellent abilities to suck and grasp objects with any shapes (flat or nonflat) and dimensions, scattered multiple objects, living bodies, super‐weight, and objects beyond the grasping range. Furthermore, the gripper features a tactile perception ability to the target objects, enabling it to distinguish objects with various dimensions and shapes, even in turbid underwater environments, demonstrating the vast application potentials in complex underwater environments.

## 2 Design and Fabrication of the Soft Gripper

### 2.1 The Glowing Sucker Octopus (*Stauroteuthis syrtensis*)

**Figure 1:**
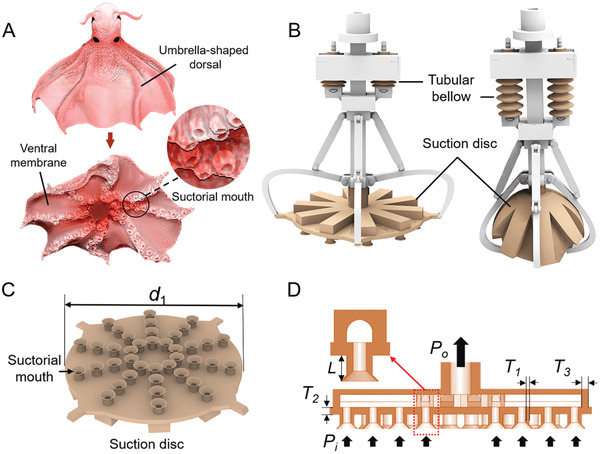
Glowing sucker octopus (*Stauroteuthis syrtensis*)‐inspired suction disc. A) Morphology structure of the *Stauroteuthis syrtensis*. Suctorial mouth arrays are distributed on the soft arms, and membranes connect the arms to form a disc. B) CAD model of the biomimetic soft gripper. The suction disc can be opened and closed under the drive of the tubular bellow. C) CAD model of the suction disc. Suctorial mouth arrays with funnel‐shaped ends are distributed on the suction disc. D) Schematics of the suction disc.

The glowing sucker octopus (*Stauroteuthis syrtensis*) features suctorial mouth arrays on a soft deformable arm with a self‐adaptive ability (**Figure** **1**A). The umbrella‐shaped dorsal membrane rolls inward to squeeze and wrap the targets with any shapes after the suckers attach to the prey. Inspired by the glowing sucker octopus (*Stauroteuthis syrtensis*) and Vampire squid's unique characteristics (such as sensing, arms, and the membrane's unique adaptive grasping structure) ,^[^ ^34^ ^]^ we developed a soft gripper (Figure 1B) with self‐adaptive and sensing abilities. The designed soft gripper has a 3D‐printed linkage mechanism, a tubular bellow inside, which can actuate a silicone‐casting soft suction disc to expand and contract. The diameter of the suction disc is *d*_1_ = 73 mm when it is at a roughly flat state without deformation. The hollow suctorial mouth arrays are evenly distributed (Figure 1C) on the suction disc. The positions of the pump connection and the fluid inlet, as in Figure 1D, are *P*_o_ and *P*_i_, respectively. All the suctorial mouths are connected to a pump to generate suction force.

### 2.2 The Fabrication of the Suction Disc

**Figure 2:**
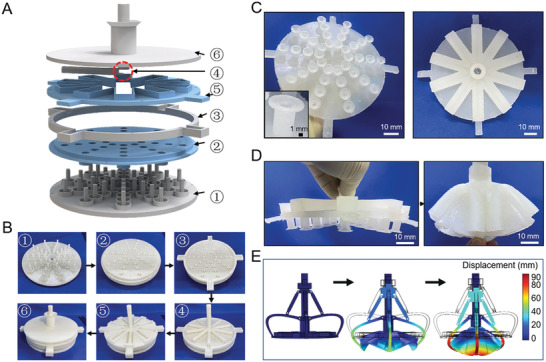
Fabrication and finite element analysis simulations of the suction disc. A) CAD model of the modular molds. The mold is designed into six parts for the integrated pouring of the suction disc. B) Schematic of the assembly of the modular mold. C) Biomimetic prototype photographs of the suction disc. The suction disc is fabricated according to the prototype of *Stauroteuthis syrtensis*. The photo of a single suctorial mouth can be seen in the picture. D) The flexibility of the suction disc. It remains unfolded in the initial state and can be closed when external force acts. E) Finite element analysis simulations of the suction disc. Hyperelastic FEM simulation on the suction disc was conducted through COMSOL and the simulation results are remarkably similar to the physical testing.

Using SolidWorks software and stereo lithography apparatus (SLA) 3D printing technology, modular molds (**Figure** **2**A and Figure S1, Supporting Information) were designed for casting the suction disc using silicone. The 3D‐printed modular molds consisted of six parts, the outer contour comprises parts ①, ②, ③, ⑤, ⑥, and Part ④ (amounts = 9) served as a support to create an internal hollow channel. The assembly process of the modular molds was presented in Figure 2B, and the silicone was poured at part ④, ⑤, and ⑥ (Figure 2B and Figure S2, Supporting Information), to obtain the suction disc (Figure 2C,D). When the suction disc is suspended in the air, it can maintain a flat structure without bending (Figure 2D). However, it had a certain degree of flexibility to achieve extensibility and adaptive grasping ability when grasping objects (Figure 2D). Hyperelastic finite element method (FEM) simulation on the suction disc was conducted through COMSOL (Figure 2E). The simulation results are remarkably similar to the physical testing. Therefore, we can use the design gripper frame combined with the suction disc to realize the grasping action.

### 2.3 The Suction Measurement of the Soft Suction Disc

**Figure 3:**
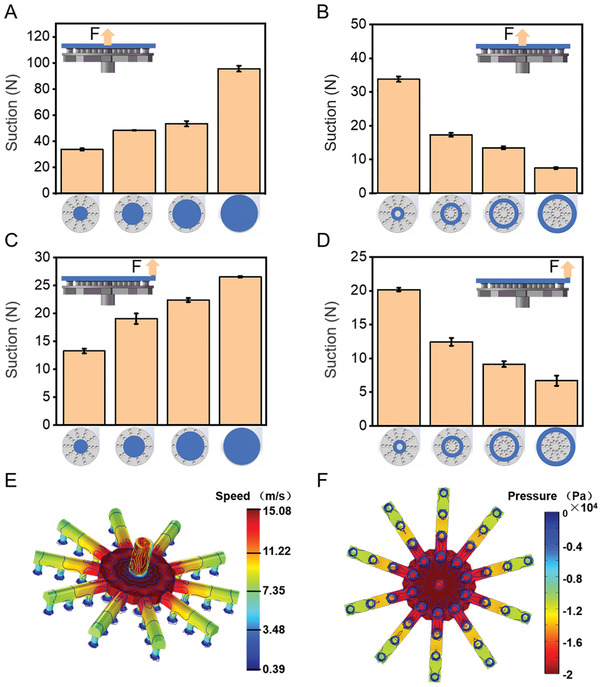
Suction test of the suction disc. A,B) When the peeling position is in the center, the suction is generated by the suction disc on A) the plane and B) the ring surface. C,D) When the peeling position is at the edge, the suction is generated by the suction disc on C) the plane and D) the ring surface. Gray represents the suction cup and blue represents the disc used to block the suction disc, which is used to test the suction. The arrow represents the position of the peeling obstacle (center or edge). E) The simulation shows the speed distribution on the inner of the suction disc. F) The simulation shows the velocity distribution inside the mouth. The inner pressure shows a decreasing trend from the inside to the outside.

The suctorial mouth arrays can produce strong suction under the air actuation generated by a submersible pump (QTX1.5‐17‐0.37L1, maximum working pressure: 318.7 KPa). The suction was characterized as the force that enabled peeling the suction disc from contacting disk or ring. When the peeling position was located at the center of the suction disc, the suction showed a gradually increasing trend when the contacting increased from the inner to the maximum plane (**Figure** **3**A).

When force *F* is applied to the center of the circular plate, the whole plate is subjected to uniform stress, and each sucker receives a uniform and equal tension. When this tension exceeds the upper limit of the suction of the sucker, the sucker falls off (Figure 3A). However, when force *F* acts on the edge of the circular plate, the stress level at the edge of the stressed disk is significantly higher than that at other places due to such stress concentration effect. Therefore, to maintain the balance of force, the suction cups in this area reach the limit suction in advance, making the whole plate fall off in advance (Figure 3C). Therefore, it can be inferred that the grasping could achieve better stability when working underwater if the captured object was directly grasped from the vertical direction. This better stability is because the suction could be higher than grasping diagonally upward.

Furthermore, we measured the change in the suction from the inner ring to the outer ring to better understand the suction distribution of the soft suction disc. It was observed that the suction for both the cases of contacting plane (Figure 3B) and ring (Figure 3D) was more significant when closer to the center of the gripper.

Besides the smooth disk or ring described above, the soft suction disc can also suck on the rougher surface (Figure S4, Supporting Information). Furthermore, the end of the tentacle can be adaptively attached to the protrusion at the bottom of the plate (Figure S4A, Supporting Information). As shown in Figure S4B in the Supporting Information, we tested the suction of the suctorial mouth on the surface with different roughness. The suction gradually decreases as the roughness increases. However, suction can still be generated on a surface with a roughness of 5000 µm, indicating that the suction disc's self‐adaptive ability to more topographically complex surfaces.

COMSOL Multiphysics‐based numerical simulations were conducted to simulate the exit velocity of the suction disc (Figure 3E,F). The pump pressure was set as *P*_o_ = 318.7 KPa and the dimension and material of the soft suction disc were consistent with the actual situation. The velocity distribution shown in Figure 3E demonstrated that the simulated suction disc's exit velocity was consistent with the experiment's measured exit velocity (14.8 m s^–1^). Furthermore, it can be seen from Figure 3F that the absolute pressure on the inner wall of the soft suction disc had a decreasing trend from the inner ring to the outer ring, indicating a decreasing suction trend from the inside to the outside, which is the same as the trend measured in the experiment (Figure 3).

### 2.4 Sensing Ability of the Soft Suction Disc

**Figure 4:**
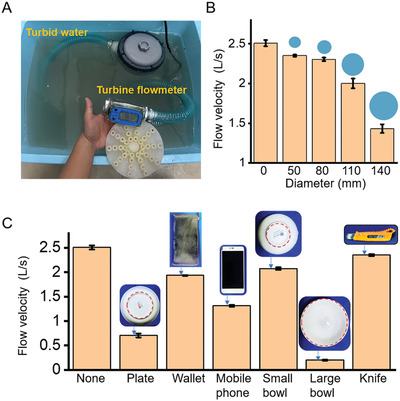
Sensing function of mouth disc in a turbid underwater environment. A) The working mechanism and working environment of mouth disc. B) When the mouth disc faces discs of different areas, the showed flow rate changes. C) Sensing test of material objects. The type of object can be distinguished based on the flow rate.

Each suctorial mouth has fluid flowing through it when it is working and the flow output from the entire soft suction disc was affected when the suctorial mouth was blocked due to contacting the target objects. Therefore, we installed a turbine flowmeter at the pump's output to utilize this flow variation phenomenon. First, the flowmeter was linked to the suction disk to monitor the flow change in real‐time to reflect the influence of the blockage of the suctorial mouths and then to distinguish the dimensions and types of the object being grasped. As shown in **Figure** **4**A, we performed the sensing test of the soft suction disc in a turbid liquid environment. Figure 4B showed the flow velocity (L s^–1^) for the grasping object diameter, and the first bar represented the flow velocity when no suctorial mouths were blocked.

Next, the relation between the flow variation and the target objects was estimated. The dimensions of an irregular object's uppermost surface (towards the suction disc) determine the number of suctorial mouths that will be engaged (to be blocked). As shown in Figure 4C, the large bowl had the largest area to be contacted by the suction disc, which exceeds the grasping range of the gripper, and its flow velocity was close to zero. The plate was contacted with a larger area and exhibited a lower flow velocity. The elongated shape of the knife had the smallest contact area. As a result, its impact on the normal flow rate was the smallest. The mobile phone, wallet, and small bowl also exhibited various flow velocities due to the various contact areas. Therefore, the suction disc can identify the dimensions of an object in an unknown space and distinguish the object when there is a priori knowledge of the types of the surrounding objects.

### 2.5 The Tubular Bellows

**Figure 5:**
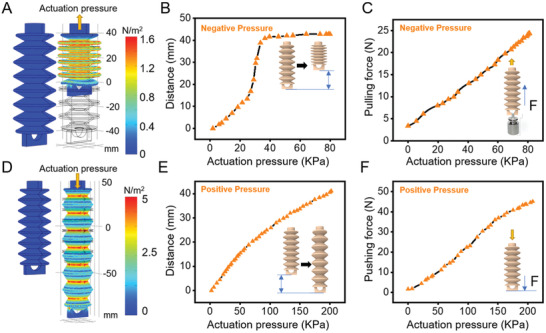
Capabilities and finite element analysis simulations of tubular bellows. A) Shrinking distance of tubular bellow under negative pressure. B) The pulling force of the tubular bellow under negative pressure. C) Hyperelastic FEM simulation of tubular bellows through COMSOL under negative pressure. D) Extend the distance of tubular bellow under positive pressure. E) The pushing force of the tubular bellow under positive pressure. F) Hyperelastic FEM simulation of tubular bellows through COMSOL under positive pressure.

Two 3D printing tubular bellows were designed as the actuators of the gripper's frame. The bellows were driven by an air pump (−80–280 KPa) to extend the suction disc by driving a slider connected to a 3D‐printed linkage mechanism to open and close the soft gripper (Movie S1, Supporting Information). COMSOL Multiphysics‐based hyperelastic FEM simulations of the tubular bellows were conducted to study the performance of the soft bellows. The simulated results (**Figure** **5**A,D) were remarkably similar to the physical testing (Figure 5B,E). The maximum shrink displacement was 42.8 mm, and the maximum extension distance was 41.1 mm. Figure 5C,F shows the push and pull generated by the tubular bellow under the pump's actuation. The maximum pulling and push force was 24.3 and 46.7 N, respectively. In particular, the tubular bellows can adjust the output force according to the characteristics of the target object

### 2.6 Assembling of the Soft Gripper

**Figure 6:**
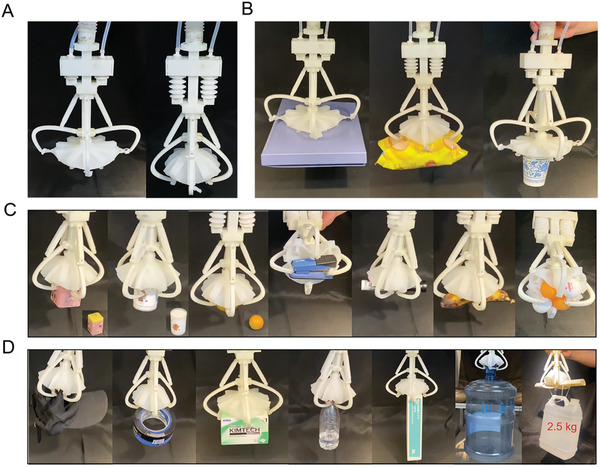
Grasping of the soft gripper in the air. A) Assembly of the soft gripper. The opening and closing of the suction disc are controlled by the expansion and contraction of the tubular bellows to realize the grasping of the object. B) The suction disc uses suction to grasp the object. The suction disc can grasp large flat objects under the negative pressure of the pump. C) Grasping of small irregular objects. Driven by the tubular bellows and assisted by the suction disc membranes, the soft gripper can hold various small objects firmly in a wrap‐around style. It is worth mentioning that the gripper could grasp multiple objects. D) Grasping of large irregular objects. Because of the large force output by the tubular bellows, the soft gripper can grasp large objects from the edge, especially an empty bulky water bottle.

In addition to the suction disc and tubular bellows, the soft gripper's structure consists of a 3D printed slider‐rod and a linkage mechanism (Figure S5, Supporting Information). A corrugated pipe connected the soft gripper to the pump and facilitated the gripper's flexible movement. The main rod was designed to be hollow for the flow to move through. A limit plate was set at the end of the hollow rod to connect the corrugated pipe. A slider consisting of two parts was driven by two paralleled tubular bellows, thus moving along the main rod. The slider consisted of two parts fixed by a cylindrical pin. The slider actuated linkage mechanisms consisting of eight links (four straight and four curved) to drive the suction disc to close and open. The head of the hollow rod was connected to the suction disc. The brace is designed to support the inside of the suction disc to prevent failure caused by the pump. Motions of the opening and close actions of the assembled soft gripper are available in **Figure** **6**A and Movie S1 in the Supporting Information.

## 3 The Grasping Experiments

### 3.1 The Grasping of the Soft Gripper in the Air

The grasping capability of the soft gripper is essential and thus needs to be investigated. Initially, the soft gripper's grasping was analyzed in the air for objects with various shapes, dimensions, and volumes (Movie S2, Supporting Information). The gripper could grasp objects with a flat surface without wrapping the suction disc by purely using suction force (negative pressure: −80 KPa) of the suctorial mouth (Figure 6B). While utilizing both suction force and the wrapping action of the suction disc, the gripper could grasp various single objects such as a square box, a bottle, a Ping‐Pong ball, a stapler, a marker, and a banana (Figure 6C). It was also worth mentioning that the gripper could grasp multiple objects simultaneously. For example, as shown in the last image in Figure 6C, the gripper grasped four Ping‐pong balls tightly. Several objects with the dimension that exceeds the grasping range were tested, as shown in Figure 6D, the gripper was able to grasp a cap, a heavy‐duty tape, a square box, a plastic bottle, a rectangular box, an empty bulky water bottle (0.8 kg) and bottle full of water (2.5 kg).

### 3.2 The Grasping of the Soft Gripper under the Water

**Figure 7:**
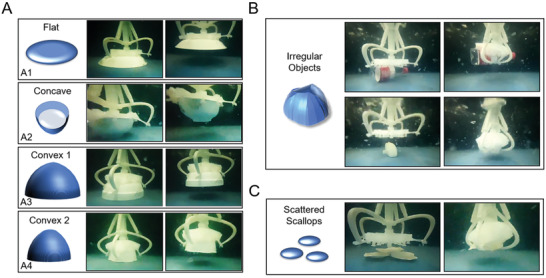
Underwater grasping performance of soft gripper. A) Grasping performance when soft gripper faces flat‐concave and convex structure objects. B) In the face of irregular objects such as water bottles and printed samples, the soft gripper can directly and firmly grasp. C) The combination of suction and adaptive grasping allows the soft gripper to grasp scattered multiple objects (scallops) at once.

Moreover, the underwater grasping ability of the soft gripper is significantly essential and was tested for different surfaces (**Figure** **7**A and Movie S3, Supporting Information). For flat objects that exceed the grasping range of the disc, the soft gripper can directly lift it by the suction generated by the suction disc (Figure 7A1). For concave objects, though the suctorial mouths were not in direct contact with the internal surface of the object, the inner space was sealed by the suction disc, thereby reducing the pressure in the enclosed cavity and generating suction to grasp objects (Figure 7A2). Convex objects that exceed the soft gripper's grasping range can be grasped by controlling the degree of curvature of the suction disc (large bowls, Figure 7A3). Objects with a greater degree of convexity can be grasped by increasing the degree of curvature of the suction disc (small bowl, Figure 7A4). In the case of irregular objects such as water bottles and printing samples (Figure 7B), the soft gripper can grasp directly and firmly. Owing to the assistance of the suction from the soft suction disc and the adaptive ability of the soft suction disc, the soft gripper can grasp multiple scattered objects (scallops) at a time to improve the grasping efficiency (Figure 7C).

**Figure 8:**
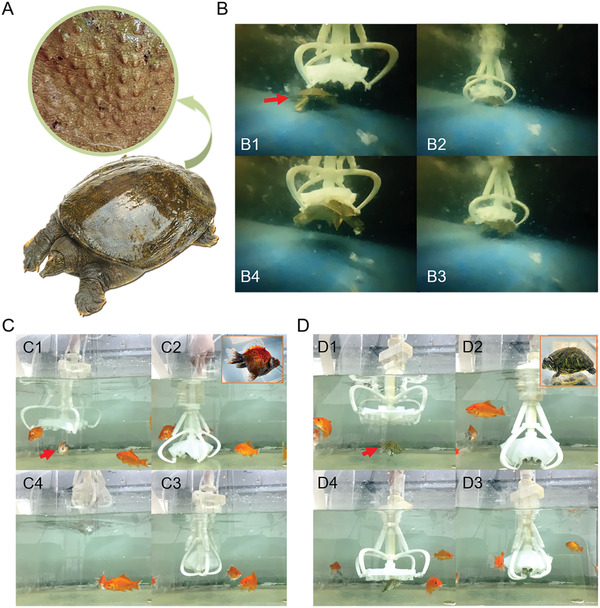
Living body grasping experiment of the soft gripper. A) The experimental object is a live turtle with small protrusions on its convex back. Its back has high roughness. B) Performance of the process of grasping moving turtle with soft grippers. Figures (B1) to (B4) show the grasping process. C) The umbrella‐shaped suction cup and suctorial mouth of the gripper can control the small fish not to escape after being caught. Figures (C1) to (C4) show the grasping process. D) The gripper uses suction to control and grasp the turtle. Figures (D1) to (D4) show the grasping process.

We also conducted a living body grasping experiment (Laboratory animal professional technical examination certificate number: TY2018466). The turtle was chosen as the experimental object, whose back was convex, and the roughness was relatively high with some small protrusions (**Figure** **8**A). The soft gripper can catch the turtle in two ways: 1) grasp the turtle by expanding and contracting the linkage mechanism and 2) use the suction disc to suck the living turtle by moving the soft gripper to touch the turtle's back (Figure 8B). Subsequently, small fish and turtle catching experiments were carried out. Thanks to the assistance of the umbrella‐shaped suction cup and nozzle of the gripper, the fish can be controlled not to escape after being caught (Figure 8C). The gripper can directly grab the turtle or grab the turtle by suction and then grab it (Figure 8D). Moreover, because the suction cup is made of soft silicone material, it can realize the nondestructive grasping of the object. All animals can swim freely in the water by opening the gripper. The living body grasping experiment demonstrated the performance of the soft gripper's catching robustness and practicality, and it did not cause any harm to those animals. All experimental demonstrations can be seen in Movie S4 in the Supporting Information.

## 4 Discussion

In this research, we developed a biologically inspired gripper based on the umbrella‐shaped dorsal and ventral membrane of the glowing sucker octopus (*Stauroteuthis syrtensis*). The fabricating process of the soft suction disc had several attractive features. Fabricating hollow connecting air channels with complex inner structures is always a considerable challenge from the perspective of traditional design and fabricating. For example, the finger structure of the fingered grippers typically relies on two methods to fabricate: 1) Dividing the finger structure into multiple parts, then conducting casting using silicone, and finally gluing the cased parts together.^[^ ^36^ ^]^ However, such a fabricating method has a potential threat to its tightness. 2) Using a fusible core mold as support for silicone casting^[^ ^37^ ^]^ will increase the cost and waste of materials. This study used the separable modular core molds, which can be assembled into a whole for silicone casting (Figure 2A,B). Such a manufacturing method ensured the airtightness of the outer wall of the suction disc, and the mold can be reused, showing potential for application in the manufacture of other types of hollow equipment.

While testing the suction of the soft gripper, a maximum suction of ≈ 100 N was generated (Figure 3A), which was three times higher than the maximum grasping force (≈ 30 N) produced by the soft fingerless gripper with the same coverage area.^[^ ^38^ ^]^ In addition to the excellent performance on smooth surfaces, the soft gripper can play a specific role in function on rough surfaces. The geometry of the flexible suction cup at the end of the suctorial mouth was designed like a funnel (Figures 1C and 2C). Thus, this funnel‐shaped geometry increases the contact area and the suction. Besides, the flexibility of the suction cup increases the adaptability to the surface of complex terrain.^[^ ^39^ ^]^ The existing soft fingerless gripper with a suction ability can only work on a relatively smooth surface.^[^ ^38^ ^]^ The glowing sucker octopus (*Stauroteuthis syrtensis*) inspired soft gripper has a more comprehensive working range. Compared with the octopus arm suction cup without pump assistance,^[^ ^39^ ^]^ the suctorial mouth can produce the same suction on the roughness of 5000 µm as the octopus arm on the roughness of 200 µm.

In addition, the hollow suctorial mouths can also be used to distinguish the dimensions and type of objects, including plates, mobile phones, wallets, small bowls, large bowls, and knives (Figure 4C). These objects represented different types: flat, strip, and 3D objects. Since the types of objects were different, they generated different flow rates through the area that the suctorial mouths can touch. Compared with the fingered grippers grasping 3D objects through the voltage generated by Triboelectric Nanogenerator^[^ ^29^, ^30^, ^31^ ^]^ to determine the degree of bending and then determine the object's dimensions, the soft gripper designed in this study can also sense the size of a flat object.

The suction disc and tubular bellows on the gripper's main framework realized the integration of the suction function, the sensing function, and the grasping function of the soft gripper. The soft griper's flexible and adaptive grasping function had better friendliness and applicability to various objects than the rigid gripper.^[^ ^40^ ^]^ The fingered grippers have inherited design limitations (multiple small 3D objects will slip through the gap when grasping and cannot grasp large flat objects) due to sparsely distributed fingers. However, the soft grippers can simultaneously grasp multiple scattered objects with the assistance of suction and the action of wrapping the suction disc around objects driven by tubular bellows. This was an absolute advantage compared to fingered grippers. In addition, since the output force of the tubular bellows was greater, as discussed above, it can generate a greater grasping force to capture the object. Compared with the fingered gripper, fingerless gripper^[^ ^38^ ^]^ driven by an air pump, the grasper relying on friction,^[^ ^18^ ^]^ and the soft doming actuator^[^ ^41^ ^]^ relying on suction, the soft gripper in this article had a more comprehensive range of grasping.

The waterproofing problem could be ignored when the tubular bellows were used underwater. Therefore, a small water pump will be fitted to the remote operated vehicle (ROV) for underwater use with the gripper. We choose to manufacture suctorial mouths of different diameters by predicting the water quality environment. Moreover, a filter system can be installed between the gripper and the pump to filter impurities to prevent impact on the pump system. The living body grasping experiment showed the application potentials of the gripper in a complex environment (Figure 8). **Table** **1** summarized the existing robotic grippers mentioned above by comparing the multimodal function, the sensing ability, the underwater application, and the grasping ability for various objects, including objects with rough surfaces, flat objects, scattered objects, and moving animals.

**Table 1:** Comparisons across the existing robotic grippers

Gripper	Multimodal	Rough surfaces	Flat objects	Scattered objects	Underwater application	Moving animals	Sensing
Our gripper	**Yes**	**Yes**	**Yes**	**Yes**	**Yes**	**Yes**	**Yes**
Finger‐like gripper^[^ ^43^ ^]^							Yes
Suction disc^[^ ^22^ ^]^		Yes	Yes		Yes		
Finger‐like gripper with suction cup^[^ ^42^ ^]^	Yes		Yes				
Multimodal, enveloping soft gripper^[^ ^19^ ^]^	Yes		Yes				
Octopus arm‐inspired gripper^[^ ^39^ ^]^	Yes	Yes	Yes				
Sea anemones inspired gripper^[^ ^18^ ^]^				Yes			
Electroadhesion gripper^[^ ^14^ ^]^	Yes		Yes				

## 5 Conclusion

This article presented a novel soft gripper inspired by the glowing sucker octopus (*Stauroteuthis syrtensis*), which can efficiently sense and grasp target objects with various dimensions and shapes. The 3D printing and modular mold casting techniques manufactured umbrella‐shaped dorsal and ventral membrane structures similar to the glowing sucker octopus (*Stauroteuthis syrtensis*). The soft gripper can output large grasping force and suction and adaptively grasp various objects such as flat objects, objects beyond the grasping range, irregular objects, scattered multiple objects, and moving bodies. It is promising that the soft gripper can use its sensing function to generate tactile sensation and conduct exploration in the turbid water environment, in which the visual perception is not available. Furthermore, the soft gripper has vast application potentials for underwater fishing and salvaging, logistics classification, and selection and placement of heterogeneous objects on the assembly line.

However, pressure leakage is a problem that needs to be considered, which will result in a decrease in the suction power of the gripper. Because some suctorial mouths are not sealed when grasping small objects. In future work, we will try to feedback the object's size through the flow meter and change the power of the pump to compensate for the pressure leakage. Or individually control each suctorial mouth: 1) Each flowmeter senses the change in flow rate and then reacts to whether it touches the object to determine the opening and closing of the mouth. 2) Add a pressure sensor at the end of the suctorial mouth to sense whether it touches an object to determine the opening and closing of the mouth.

## 6 Experimental Section

### Design of the Modular Mold

The modular mold was designed by Solidworks. The designed computer aided design (CAD) models were sliced with Materialise Magics Software and printed by SLA 3D printer. The printing material was photosensitive resin. The printer (SLA550) and printing materials were purchased from Zrapid Tech Co., Ltd. The mold was divided into six parts (Figure S1, Supporting Information) named ①, ②, ③, ⑤, and ⑥, which were used to form the outer contour. The core mold ④ was used as a support to form an internal hollow channel. Molds ① and ② were responsible for forming the funnel‐shaped suctorial mouths and the hollow channels inside. Each suctorial mouth had a length of *L* = 8 mm, the inner diameter of the suctorial mouth *d* = 2.25 mm, a wall thickness of *T*_1_ = 1.75 mm, and the geometries of the funnel at the end (Figure 1D). Molds ① and ④ were assembled to form an internal through channel (Figure S1, Supporting Information). The molds ②, ③, ⑤, and ⑥ form the disc and the channel wall. The thickness of the disc and the wall thickness of the channel were both *T*_2_ = *T*_3_ = 4 mm, the inner height of the channel *h *= 7 mm (Figure 1D), the top was a dome, and the arc radius was 4 mm (Figure 1D). After repeated tests, when the channel wall thickness was set to 4 mm (Figure 1D), there was no collapse in the internal passage of the mouth disc, which can exert the maximum function of the pump and did not affect the flexibility of the soft gripper for adaptive grasping. In addition, the top of the internal channel was designed in a circular arc shape (Figure 1D) to prevent stress concentration and withstand more significant water pressure. Casting ears on the mouth disc were used to facilitate the combination of the mouth disc and the gripper frame (Figure 2A–D).

### Fabrication of Suction Disc

The silicone used for casting the soft suction disc was purchased from Beijing Sanxin Jingde Co., Ltd., with a shore hardness of 15 A. A layer of petroleum jelly (by Vaseline) was coated with all mold modules’ surfaces, which were in contact with the silicone, thus facilitating the disassembly of the mold by avoiding sticking. Vaseline was purchased from Nanchang Baiyun Pharmaceutical Co., Ltd. Assemble molds ①, ②, ③, and ④ in sequence (Figure 2B and Figure S3, Supporting Information) and pour silicone. After assembling the No. 5 mold, continue to pour the silicone. After putting the remaining silicone and the poured mold into a vacuum dryer and vacuuming for 30 min, the No. 6 mold is assembled, and the remaining silicone is poured. Then the mold is fixed and sealed. The soft mouth disc was integrally cast with silicone, weight *W* = 170 g, and mouth disc diameter *d*_1_ = 73 mm. When disassembling the mold (Figure S3, Supporting Information), follow the order of ⑥, ⑤, ③, ①, and ② to disassemble the mold in turn, and finally take out all the core molds from the channel opening, and the soft mouth disc was completed.

### Measurement of Suction

Self‐built measuring equipment is used for suction measurement of suction cups on discs and rings. First, a rigid stent is equipped inside the suction cup. Then the suction cup connected to the pump is fixed underwater by a bench vice. The steel frame structure fixed the linear actuator directly above the suction cup. Afterward, the linear actuator, tension meter, and disk (or ring) were assembled vertically using the connecting mechanism made by 3D printing. After the suction cup is in contact with the disk (or ring), the linear actuator is actuated to separate it. Moreover, the video recorder records the reading change of the Tension meter currently. Finally, adjust the position of the bench vice to obtain suction at different peeling points (Figure S6, Supporting Information).

### Fabrication of Tubular Bellow

Tubular bellow was designed by SolidWorks, with a wall thickness of 1.5 mm. The designed tubular bellow was imported into the software CURA to generate printable slices in the G‐Code format, a standard procedure for 3D printers. The 3D printer (Polarbear S5) was purchased from Polar Bear Technology Co., Ltd. The printing temperature is 220 ℃, the printing speed is 4 mm s^–1^, the extruder speed is 5.5 mm s^–1^, the printing layer height is 1.4 mm, and the diameter of the extrusion nozzle is 0.25 mm. The wire used for printing is TPU (Thermoplastic polyurethane) material with a diameter of 1.75 mm and a Shore hardness of 85 A.

### FEM Simulations

The working principle of the designed soft gripper, as in Figure 2E, was validated prior to fabrication by simulating the linkage mechanism actuating expansion and contraction action of the soft suction disc in COMSOL Multiphysics (multibody dynamics simulations). Silicon material with five‐parameter Mooney–Rivlin model and epoxy material (density ≈ 980 kg m^–3^, Young's modulus ≈ 2.79E9 Pa, and Poisson's ratio ≈ 0.38) were defined to the suction disc and the linkage mechanism, respectively. The designed soft gripper's elements were defined as a multibody system, in which rigid‐type joints were defined between adjacent rods. In addition, the slider linked to the linkage mechanism was defined as a predefined displacement of 42 mm, which equaled the actual maximum displacement of the soft tubular bellow. Further, a predefined finer physical‐controlled mesh was used to generate the mesh for the whole gripper. Finally, a transient simulation lasting 1 s with a fully coupled solver was conducted to solve the deformation of the suction disc and motions of the linkage mechanism and the slider (Figure 2E). Hyperelastic FEM simulations were conducted in COMSOL Multiphysics to observe the change in length of the tubular bellows under different air pressure without spending more than 15 h to print the model and conduct testing (Figure 5C,F), which significantly reduced the prototyping time. The 85A TPU material (NinjaFlex, from NinjaTek, USA) with a five parameters Mooney–Rivlin model^[^ ^42^ ^]^ was defined as the tubular bellows. The air pressure was simulated by applying equal boundary loads on all the cavity surfaces of tubular bellows. By changing the load, the morphing of the tubular bellows when pressurizing and vacuuming with different pressures was simulated and observed.

## Conflict of Interest

The authors declare no conflict of interest.

## Supporting information

Supporting Information

Supplemental Movie 1

Supplemental Movie 2

Supplemental Movie 3

Supplemental Movie 4

## Data Availability

The data that support the findings of this study are available from the corresponding author upon reasonable request.
