# Supplementary material for: Glowing Sucker Octopus (Stauroteuthis syrtensis)‐Inspired Soft Robotic Gripper for Underwater Self‐Adaptive Grasping and Sensing
Source: Adv Sci (Weinh). 2022 Apr 7;9(17):2104382. doi: 10.1002/advs.202104382 (PMC9189663; doi:10.1002/advs.202104382)
Supplement: Supplementary file 1 — Supporting Information [file ADVS-9-2104382-s002.pdf]

ADVANCED SCIENCE
Open Access

Supporting Information

for *Adv. Sci.*, DOI 10.1002/advs.202104382

Glowing Sucker Octopus (*Stauroteuthis syrtensis*)-Inspired Soft Robotic Gripper for Underwater Self-Adaptive Grasping and Sensing

Mingxin Wu, Xingwen Zheng, Ruosi Liu, Ningzhe Hou, Waqar Hussain Afridi, Rahdar Hussain Afridi, Xin Guo, Jianing Wu, Chen Wang and Guangming Xie**

Supporting information

Glowing sucker octopus (*Stauroteuthis syrtensis*)-inspired soft robotic gripper for underwater self-adaptive grasping and sensing

Mingxin Wu[1], Xingwen Zheng[1,2,] *, Ruosi Liu[1], Ningzhe Hou[3],

Waqar Hussain Afridi[1], Rahdar Hussain Afridi[1], Xin Guo[1],

Jianing Wu[4], Chen Wang[1], Guangming Xie[1,5,6,7] *

[1] State Key Laboratory for Turbulence and Complex Systems, Intelligent Biomimetic Design Lab, College of Engineering, Peking University, Beijing, 100871, People's Republic of China

[2] Advanced Production Engineering, Engineering, and Technology Institute Groningen, Faculty of Science and Engineering, University of Groningen, Groningen 9747AG, The Netherlands

[3] Department of Bioengineering, Imperial College London, South Kensington, London SW7 2AZ, United Kingdom

[4] School of Aeronautics and Astronautics, Sun Yat-Sen University, Guangzhou, 510006, People's Republic of China

[5] Southern Marine Science and Engineering Guangdong Laboratory (Guangzhou)

[6] Peng Cheng Laboratory, Shenzhen, 518055, China.

[7] Institute of Ocean Research, Peking University, Beijing, 100871, China.

* Corresponding authors: Guangming Xie (xiegming@pku.edu.cn) and Xingwen Zheng (zhengxingwen@pku.edu.cn)

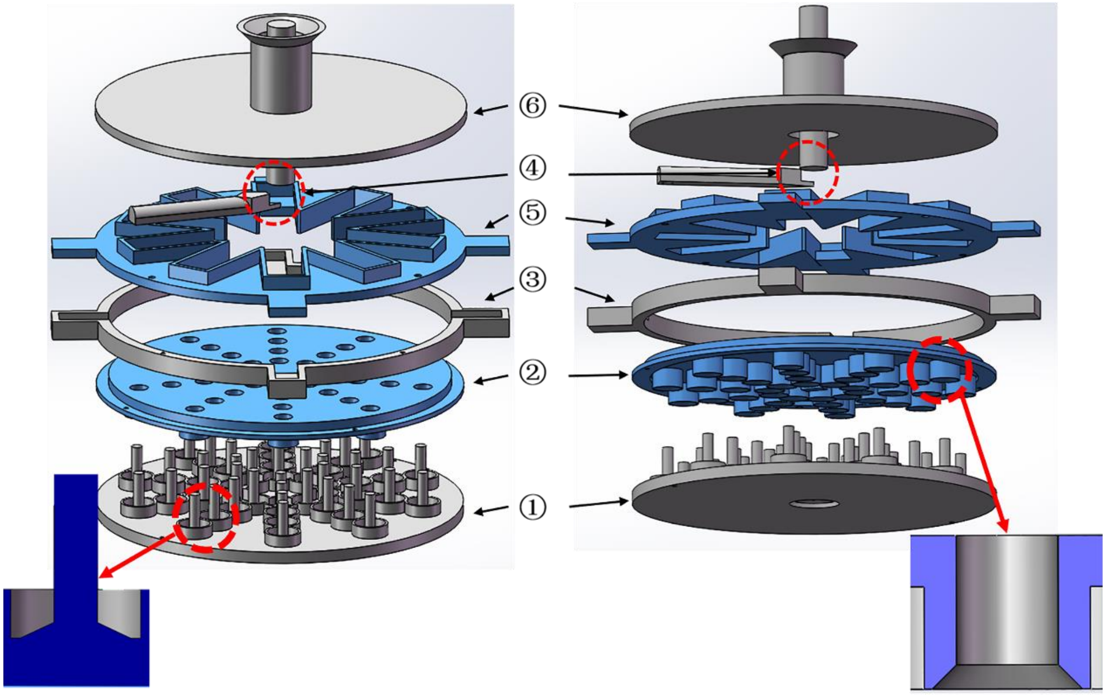

Figure S1. CAD model of the modular molds. The mold is divided into 6 parts: 1, 2, 3, 5, 6 are used to form the outer contour, and the core mold 4 is used as a support to form an internal hollow channel. Mold No. 1 is used to form the funnel-shaped tentacles.

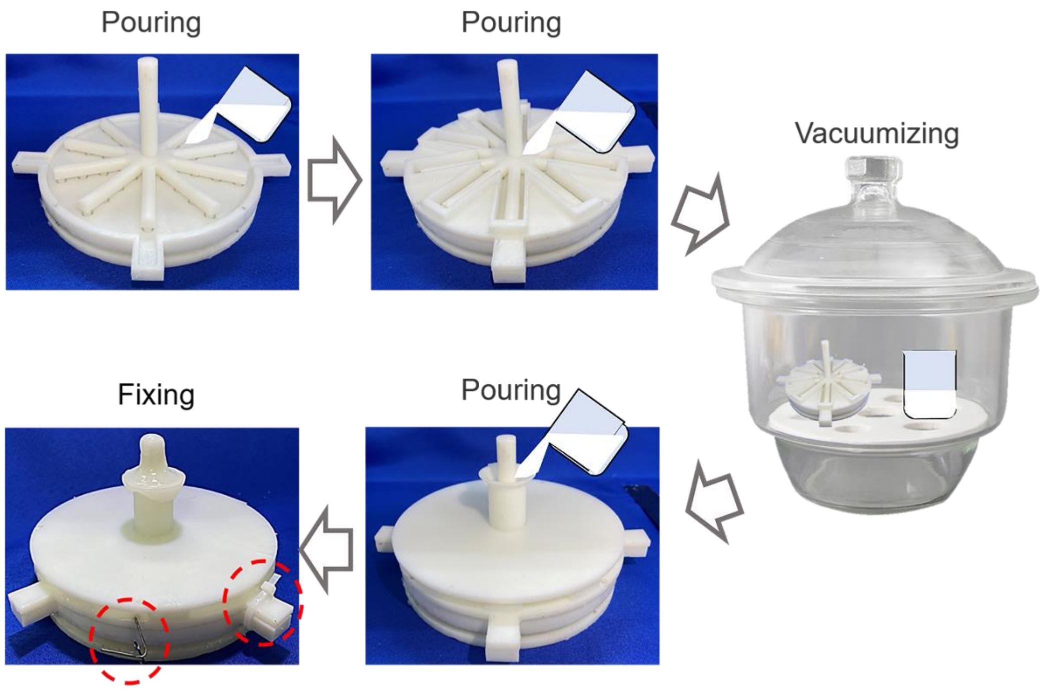

Figure S2. The casting process of the mouth disc. Assemble 1-2-3-4 molds in sequence and pour silicone. Continue to pour the silicone after assembling the No. 5 mold. After putting the remaining silicone and the poured mold into a vacuum dryer and vacuuming for 30 minutes, the No. 6 mold is assembled, and the remaining silicone is poured, and then the mold is fixed and sealed.

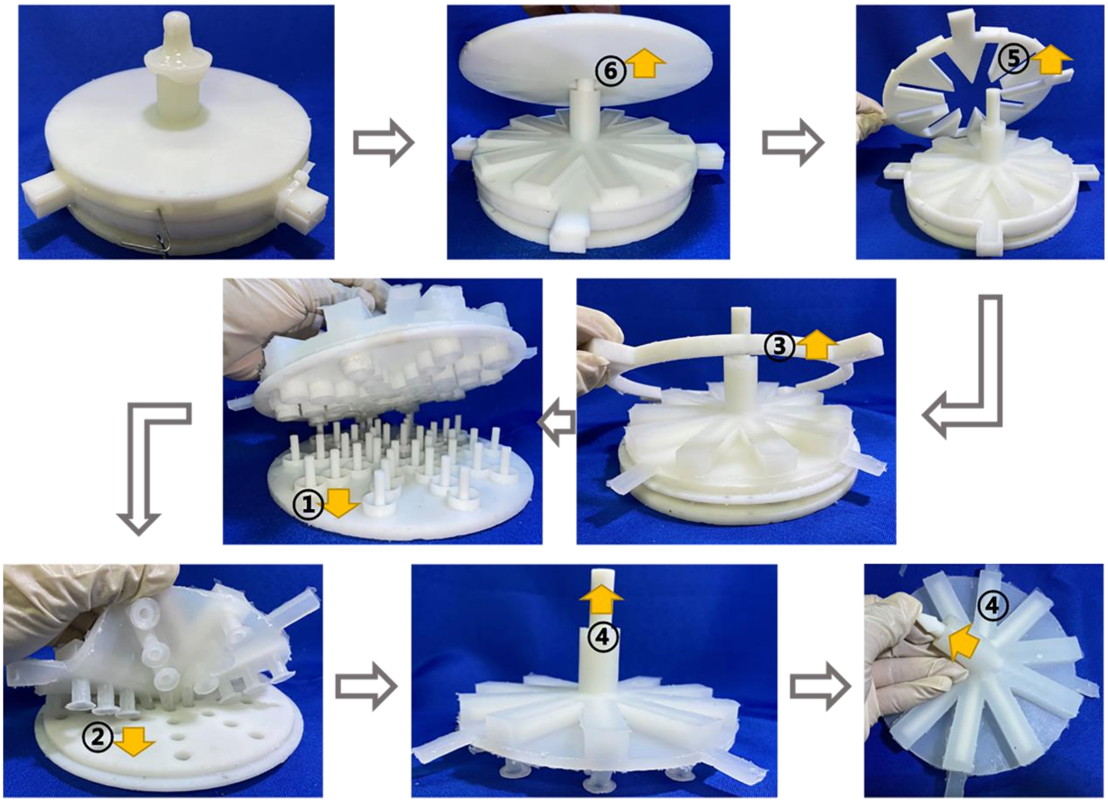

Figure S3. Demoulding process. When disassembling the mold, follow the order of 6-5-3-1-2 to disassemble the mold in turn, and finally take out all the core molds from the channel opening, and the soft mouth disc was completed.

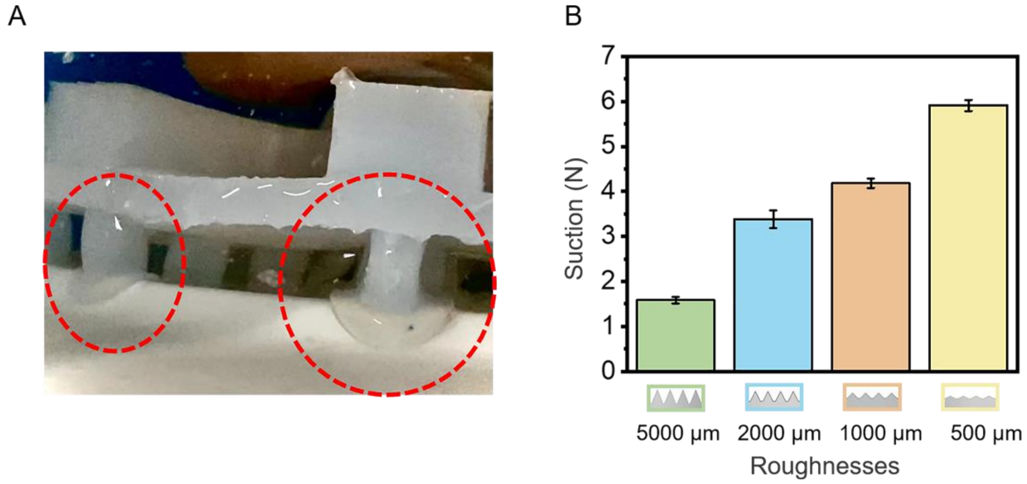

Figure S4. The compliance of the end of the tentacles can be adapted to the surface with complex morphology. (A) The end of the tentacle can be adaptively attached to the protrusion at the bottom of the plate. **(B)** The suction of the mouth disc on the surface of different roughness. Surfaces with different roughness are prepared by a light-curing 3D printer.

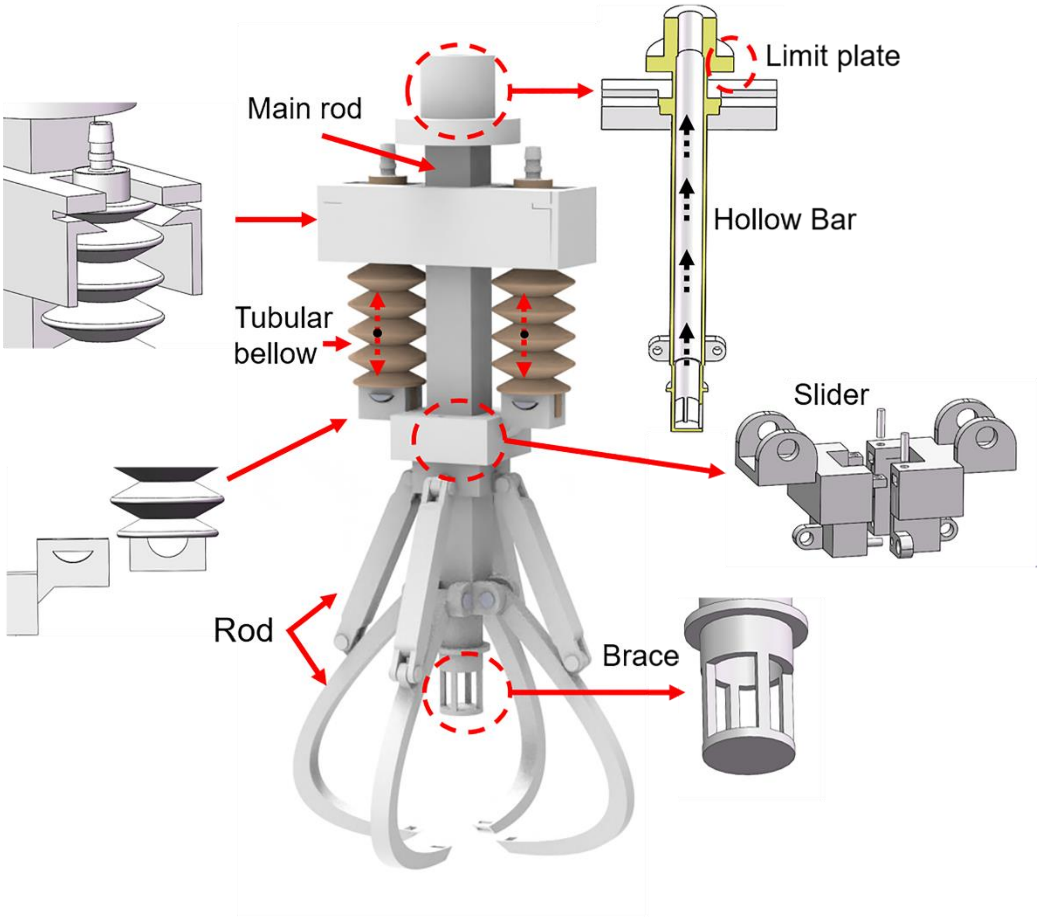

Figure S5. Gripper designed for the soft gripper, including hollow bar, main rod, slider, stent, limit plate, and electric push rods.
